# Sharing Human-Generated Observations by Integrating HMI and the Semantic Sensor Web

**DOI:** 10.3390/s120506307

**Published:** 2012-05-11

**Authors:** Álvaro Sigüenza, David Díaz-Pardo, Jesús Bernat, Vasile Vancea, José Luis Blanco, David Conejero, Luis Hernández Gómez

**Affiliations:** 1 ETSI Telecomunicación, Universidad Politécnica de Madrid, Avenida Complutense 30, E-28040 Madrid, Spain; E-Mails: dpardo@gaps.ssr.upm.es (D.D.-P.); mr.vasilevancea@yahoo.com (V.V.); jlblanco@gaps.ssr.upm.es (J.L.B.); luisalfonso.hernandez@upm.es (L.H.G.); 2 Telefónica Investigación y Desarrollo, Distrito C. Edificio Oeste 1, Ronda de la Comunicación, s/n, 28050 Madrid, Spain; E-Mails: bernat@tid.es (J.B.); dco@tid.es (D.C.)

**Keywords:** connected objects, connected cars, human-generated observations, Human-Machine Interaction, Sensor Web, Semantic Sensor Web

## Abstract

Current “Internet of Things” concepts point to a future where connected objects gather meaningful information about their environment and share it with other objects and people. In particular, objects embedding Human Machine Interaction (HMI), such as mobile devices and, increasingly, connected vehicles, home appliances, urban interactive infrastructures, *etc*., may not only be conceived as sources of sensor information, but, through interaction with their users, they can also produce highly valuable context-aware human-generated observations. We believe that the great promise offered by combining and sharing all of the different sources of information available can be realized through the integration of HMI and Semantic Sensor Web technologies. This paper presents a technological framework that harmonizes two of the most influential HMI and Sensor Web initiatives: the W3C's Multimodal Architecture and Interfaces (MMI) and the Open Geospatial Consortium (OGC) Sensor Web Enablement (SWE) with its semantic extension, respectively. Although the proposed framework is general enough to be applied in a variety of connected objects integrating HMI, a particular development is presented for a connected car scenario where drivers' observations about the traffic or their environment are shared across the Semantic Sensor Web. For implementation and evaluation purposes an on-board OSGi (Open Services Gateway Initiative) architecture was built, integrating several available HMI, Sensor Web and Semantic Web technologies. A technical performance test and a conceptual validation of the scenario with potential users are reported, with results suggesting the approach is sound.

## 1. Introduction

The current evolution of ubiquitous computing and information networks is rapidly merging the physical and the digital worlds enabling the ideation and development of a new generation of intelligent applications as eHealth, Logistics, Intelligent Transportation, Environmental Monitoring, Smart Grids, Smart Metering or Home Automation. This scenario, seminal in Mark Weiser's Ubiquitous Computing work [1] and now evolving into the “Internet of Things” [2] concept, points toward a future in which many objects around us will be able to acquire meaningful information about their environment and communicate it to other objects and to people.

Among this universe of interconnected objects, those embedding Human-Machine Interaction (HMI) technologies, such as mobile phones, connected vehicles, home appliances, smart buildings, interactive urban infrastructures, *etc*., can play an important role as they can be aware of real-world information and, at the same time, provide enriched information to other users, objects or applications. Data could come from human (social networks, monitoring systems, interactive devices), or from machine input (e.g., different Sensor Networks), and the HMI is the connection between these two sources.

Sensor and Actuator Networks (SANs) are becoming an inexhaustible source of real world information, so the Sensor Web term is being used to describe a middleware between sensors and applications: “Web accessible sensor networks and archived sensor data that can be discovered and accessed using standard protocols and application programming interfaces” [3]. An emerging number of Sensor Web portals, such as Sensorpedia [4], SensorMap [5], SensorBase [6] or Pachube [7], are currently being developed to enable users to upload and share sensor data. One of the most influential Sensor Web initiatives is the Sensor Web Enablement (SWE) of the Open Geospatial Consortium (OGC). The SWE [8] is defining a set of standards to develop “an infrastructure which enables an interoperable usage of sensor resources by enabling their discovery, access, tasking, as well as eventing and alerting within the Sensor Web in a standardized way”.

Further efforts to improve interoperability of a world of heterogeneous and geographically dispersed interconnected SANs include the proposal of a Semantic Sensor Web [9,10]. The Semantic Sensor Web brings Semantic Web technologies to annotate sensor data making it easier for different applications to extract homogeneous interpretations of them.

To progress towards a full harmonization between HMI systems and the Sensor Web, advancements are needed in two fundamental areas: the integration and accessibility of a growing number of heterogeneous sensor data into HMI systems, and new mechanisms that allow sharing real-world information provided by users of connected objects into the Sensor Web or the Semantic Sensor Web.

In our previous research [11] we have presented some contributions to the first issue, so in this paper we will try to contribute to the second one. In particular we will present our results based on our activities in the Mobility for Advanced Transport Networks (MARTA) project [12], a Spanish publicly funded project where several context-aware interactive services were designed and implemented for In-Vehicle Information Systems (IVIS) and Advanced Driver Assistance Systems (ADAS). It is important to point out that, as stated before, we believe that the proposed framework for integrating HMI and Semantic Sensor Web principles and technologies is general enough and could be applied in a variety of scenarios featuring mobile devices, multimedia and home appliances, urban interactive infrastructures, *etc*.

Nevertheless, in order to make the presentation of the proposed framework clearer, in this paper we will focus on a scenario where a driver of a connected car provides, through interaction with an in-vehicle HMI system, contextual information that can be valuable for other applications. For example, the driver may detect potential dangers on the road (ice-patches, pedestrians, *etc*.), or certain traffic conditions (accidents or congestions) or environmental conditions (dense fog or heavy rain). Then, by interacting with an in-vehicle HMI system, (s)he can make this contextual information available to other interested applications (e.g., a Road Safety Authority or other HMI systems in surrounding connected vehicles). In the manner of recent proposals such as the Human Sensor Web [13], these pieces of contextual information that user of the connected object (*i.e.*, the driver) provides will be referred to here as *human-generated observations*.

**Figure 1.**
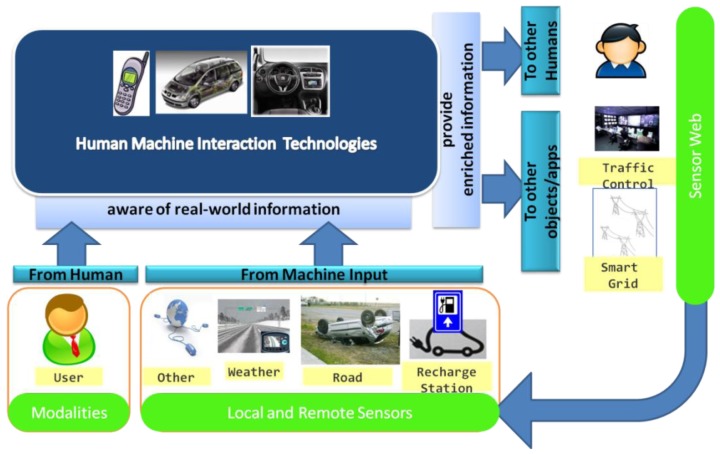
In-vehicle HMI system for connected cars.

Future in-car interaction scenarios must be considered not as simple “local” driver-system interfaces, but, as Figure 1 illustrates, as complex systems. HMI systems for connected cars have to manage, not only different driver's interaction modalities (speech–microphones and loudspeakers; vision–displays; haptic–knobs, buttons, touch screen; *etc*.), but also local and remote sensor information. As shown in Figure 1, context-aware HMI systems can be regarded as systems that use sensor data and user inputs to interact with applications, but at the same time HMIs may be regarded as sensing systems capable of producing real-world information for the Sensor Web. This capability of using HMI systems embedded in a connected object to publish information into the Sensor Web could be related, either to measurements from its local sensors (attached to the object), or to data directly provided by her user. In [14] we discussed some of the main issues when using HMI systems to process and publish local sensor data. In this paper we will address those related to the publication of user-generated observations.

In this work we will also rely on the design principles proposed by the W3C's Multimodal Architecture and Interfaces (MMI) [15]. Following these principles we will discuss the design of in-vehicle context-aware multimodal HMI systems capable of collecting driver's information reporting observations on different road, traffic or environmental situations, and generate semantic representations of them.

The rest of the paper is organized as follows: Section 2 presents related research. Section 3 describes the design of in-vehicle HMI systems to collect driver-generated observations following the principles of the W3C's MMI architecture instantiated on an OSGi framework. The semantic annotation of driver-generated observations and their publication in the Semantic Sensor Web are discussed in Section 4. Section 5 presents our experimental set-up, implemented on an on-board unit of a connected car. Performance analyses and a concept validation study are described in Section 6. Finally, conclusions and future work are discussed in Section 7.

## 2. Related Work

In-vehicle context-aware HMI systems and the more recent conceptions of user-generated sensors or the Human Sensor Web [13,16] are two research areas closely related to the work in this paper. Recent research on context-aware HMI systems in general, and in-vehicle interactive systems in particular, has sought to ensure that they are able function in highly heterogeneous environments, adapting to all kinds of situations and contexts, always giving correct and safe feedback to their users ([17–19]). Information services embedded in HMI systems have to manage a common representation of the user (identifying his mood state, needs and preferences) and the contextual situation coming from a variety of heterogeneous sources. In order to integrate this data in a homogeneous manner, some approaches, such as the one presented in [20], have already made use of Semantic Web technologies to define a model of contextual information composed of several independent ontologies, mainly to represent users, devices, environment and services.

In HMI vehicle scenarios, integrating both multimodal interaction and context for in-vehicle applications has also been addressed, and a common approach [19] is to consider three independent domains: driver, vehicle and environment. However, most of the research in context-aware multimodal HMI systems in vehicles has been more focused on how to manage high-level representations of context than on the integration with the underlying infrastructures providing sensor data. Only few approaches, such as the work presented in [21] for an in-car OSGi framework, have addressed the design of HMI systems including the management of different car components. Nevertheless, these studies only take into account data from local sensors (attached to the car) and do not consider the access or sharing (publication) of sensor data through the Internet.

The research presented in this paper can be also related to emergent concepts of user-generated sensors or human observations (descriptions of real-world phenomena), that are different from those of human sensor observations (particular sensors carried by or attached to humans). The seminal work in [13] presents the Human Sensor Web vision as “an effort for creating and sharing human observations as well as sensor observations on the Web”, and presents an example of establishing a noise mapping community. According to this vision, future systems will use different types of observations: conventional sensor data, human sensed observations (e.g., vocal, image or text) and human collected data (sensors carried by humans, like smart phones or other personal devices), and will integrate them into the Human Sensor Web [16]. The work in [13] also identifies some challenges to realize the Human Sensor Web. The most persistent challenges are guaranteeing the accuracy of the data, resolving personal privacy issues and answering the fundamental question of how collective intelligence can improve on conventional methods [22]. In a similar direction, in our work we will discuss preliminary approximations to using semantic representations–which are already being used to represent sensor data–to describe human observations, and we will explore the use of the Semantic Sensor Web principles for publishing and accessing them.

## 3. HMI Systems to Collect Driver Observations

As we stated before, developing in-vehicle HMI systems requires not only the integration of the driver's input/output information (e.g., speech, touch, graphic displays, *etc*.), but also the proper management of data provided from different sensor sources: from the car (e.g., speed, wheel traction), the driver (e.g., mood, fatigue), and environment (road, traffic, weather, *etc*.) [23]. The HMI designer typically needs to interpret the sensed data in order to identify situations that are either of direct interest to the driver, or which will help to shape communication strategies that are appropriate for each situation.

**Figure 2.**
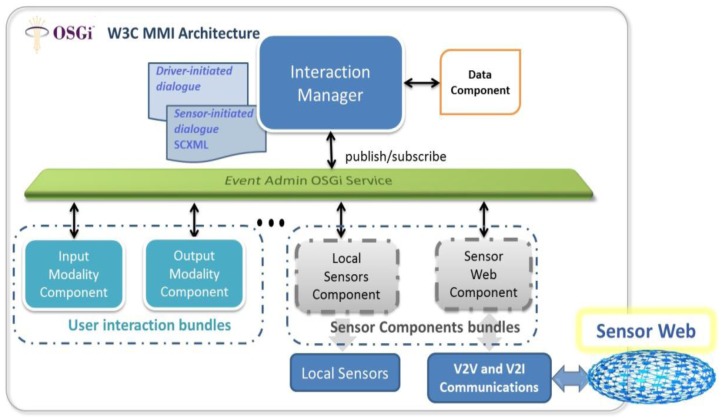
In-vehicle HMI system to collect driver observations, following the W3C MMI Architecture.

The W3C is in the process of defining an architecture recommendation for the design of multimodal interfaces: the MMI Reference Architecture [15]. Major components in the W3C MMI architecture, represented in Figure 2, are the Input and Output Modality Components, which handle the information coming in from and going out to the human user, and the Interaction Manager, which coordinates the flow of the communication in the different modalities and decides the overall communication strategy in response to successive inputs from the user. The MMI architecture also considers two important elements: (1) a data component which stores the data that the Interaction Manager needs to perform its functions; and (2) an event-based communication layer to carry events between the modality components and the Interaction Manager.

This standardized reference architecture provides a very attractive framework for dealing with the high complexity of designing HMI systems for a variety of connected objects such as connected vehicles. In our experimental implementation, that will be detailed in Section 5, an OSGi framework [24] was used to instantiate an embedded W3C MMI architecture into a connected car. OSGi is a Java-based service platform that allows applications to be developed from small, reusable and collaborative components called bundles. The main components in the MMI Architecture (*i.e.*, Interaction Manager, and Input/Output Modality Components) can be implemented as OSGi bundles. The platform also provides an EventAdmin OSGi Service bundle as a standard way of dealing with events in the OSGi Environment using the publish/subscribe model. Therefore, this event management capability in OSGi can represent the event-based communication layer in the W3C MMI architecture. The mapping between these OSGi capabilities and the MMI architecture is illustrated in Figure 2.

Extending this basic HMI architecture to include information from different sensor sources is rather straightforward. Information from both local sensors (attached to the car or connected object) and remote sensors (e.g., from the Sensor Web) can be directly accessed by developing specific bundles acting as “Sensor Components” between the sensor providers and the HMI Interaction Manager (see the Local Sensor Component and the Sensor Web Component in Figure 2).

Obviously, to have access to remote sensors (*i.e.*, the Sensor Web Component) the OSGi framework must also include a communication infrastructure—for example, in the case of a connected car, supporting V2V (vehicle to vehicle) and V2I (vehicle to infrastructure) communications, or just communication capabilities through in-car nomadic devices, such as the driver's mobile phone (OSGi is also a technology suitable for integration into mobile phones and other connected objects).

Inside the W3C MMI architecture, as in any HMI system, a key component is the Interaction Manager. The Interaction Manager receives ordered sequences of events and data from the different Components (both from the user and sensor sources) and decides what to do with them. Events may be for the Interaction Manager's own consumption, they may be forwarded to other components or they may result in the generation of new events or data by the Interaction Manager. For the purpose of designing flexible and easily configurable Interaction Managers the W3C is developing SCXML (State Chart eXtensible Markup Language) [25], a generic event-based state-machine execution environment based on Harel statecharts [26]. Statecharts are extensions of conventional finite state machines, with additional properties that lend themselves to describing complex control mechanisms in reactive systems in which it is necessary to coordinate components of diverse nature. SCXML is being proposed by the W3C as a major candidate language to control interaction flow in Human-Machine Interactive systems (HMIs). It is being considered for future interactive speech systems, in W3C VoiceXML 3.0 [27], as well as for multimodal systems [15]. As we have presented in previous work [28], SCXML can be also very useful for combining user input information and sensor information.

In this work we have implemented an SCXML-based Interaction Manager controlling the data exchanges with the driver (see the details in Section 5). Driver input information is obtained using speech recognition controlled with a push-to-talk button on the steering wheel, and output information is provided through text-to-speech synthesis and a visual display. Two different models of spoken dialogue interaction have been implemented for collecting driver observations: *sensor-initiated* and *driver-initiated*. Sensor-initiated dialogue starts automatically when a sensor detects a possibly relevant situation. The sensor-initiated dialogue is a rather simple one since the HMI system has only to ask the driver to confirm (using yes/no expressions) the particular sensor-detected situation or observation. A system-generated interaction might follow a structure such as:

SYSTEM: The car's sensors are detecting limited visibility. Please confirm whether there is fog or a dust cloud.
USER: Fog.
SYSTEM: Thank you for confirming the presence of fog. Security systems have been adjusted accordingly.

In driver-initiated dialogue, the dialogue is started by the driver, using a specific button in the steering wheel, when she observes what she believes is a relevant situation; for example, entering a densely foggy area, or upon seeing a tree fallen across one of the lanes of the road. An example dialogue might be:

USER: There is a fallen tree on the right lane.
SYSTEM: Tree on right lane. Thank you for informing. The observation has been relayed to Traffic Control.

Driver-initiated dialogue presents a more challenging situation because the number of different kinds of observations a driver can report can be potentially very high. Furthermore, the spontaneous language she can use can be very rich and varied, requiring Natural Language Processing capabilities not implemented in our OSGi framework. To limit these problems, in our implementation driver-initiated interaction has been restricted to a menu-based dialogue. Once the driver decides to report an observation, she has to follow a system-directed dialogue offering a limited set of possible observations. In order to avoid speech recognition errors, which can lead to unsafe driving situations [29], the number of different observations has been limited to 16, arranged into two sub-menu levels. In the first level the driver has to choose the category of her observation—road, traffic or environment, and in the second level she has to select the particular observation in the selected category. Some results from a preliminary usability evaluation of the test scenario are discussed in Section 6. The upcoming tests described in Section 6 will address problem situations such as those derived from speech recognition errors, with the aim to gain an understanding on the interaction effects between the (simulated) driving task and the dialogue task.

Finally, it is important to point out that, apart from the difficulties in designing robust and safe spoken dialogue strategies to collect human-generated observations, an important challenge, not addressed in our work, is how to provide a confidence level on the quality of the information the driver is reporting. Some strategies already in use in social networks could be explored, such as ratings of particularly reliable users or matching for coincident observations [30].

## 4. Publishing Driver Observations into the Semantic Sensor Web

Once a driver-generated observation has been collected through a driver-initiated or a sensor-initiated dialogue, the Interaction Manager has to start a procedure to make it available into the Sensor Web. Two main steps are required: (1) to provide a homogeneous representation for the human-generated observation; and (2) to drive a mechanism to publish it in the Sensor Web.

As mentioned in the Introduction (Section 1), there is currently an emergence of Sensor Web portals (*i.e.*, Pachube, Sensorpedia, *etc*.), and they could be considered for publishing human-generated observations. Another family of resources that could be explored for these purposes are Social Network infrastructures, such as text-based posts (e.g., Twitter [31]).

In this work we will explore OGC SWE principles [8], as they constitute one of the most mature and active proposals in the field. Nevertheless, specifying the requirements for publishing driver-generated observations using current SWE standards is far from trivial. Here are some major points that must be taken into account:

- First, it would be necessary to describe the in-vehicle HMI system as a sensing system using SensorML (Sensor Model Language) [8]. SensorML is the OGC SWE language used to describe different types of sensors and sensor systems, from simple to complex, such as earth observing satellites or, in our case, a driver-observer.
- Then, this observation-generating entity must be registered into an OGC Catalog Service (CS-W) [4], so that its observations can be discovered by other applications.
- Driver observations should be represented using the O&M (Observation & Measurement) language [8]. O&M defines a domain-independent conceptual model for the representation of–spatiotemporal– sensed data.
- Finally, the human-generated sensor resources have to be registered, and made discoverable and accessible using a set of basic Web Services, such as the Sensor Observation Service (SOS) [8] (SWE only standardizes their interfaces).

Due to the difficulty of addressing the above points in our in-vehicle environment, in this work we have sought to drift toward the recent initiative of blending the Sensor Web with Semantic Web technologies, into what is referred to as the Semantic Sensor Web [9,10]. Notwithstanding the fact that, as stated in the position paper presented in [32], it can be hard to measure how successful these recent initiatives are, we will explore the use of URI-based (Uniform Resource Identifier) descriptions of human-generated observations encoded using the Resource Description Framework (RDF), as this is an accepted Semantic Web standard [33]. This will facilitate building many applications such as Web mashups and, as we will discuss and illustrate in Section 5, by adopting Linked Data principles (Linked Sensor Data [32]), “to use URIs as reference for look-up as well as RDF and SPARQL (SPARQL Protocol and RDF Query Language) for storage, access, and querying”.

So far, adopting what we can call Semantic Sensor Web principles, the following sub-sections will discuss how to describe, store and access the HMI-collected driver-generated observations.

### 4.1. Semantic Description of Driver-Generated Observations

Annotating human-generated observations using semantic models (*i.e.*, RDF and OWL) can provide important benefits over other schemes:

- It offers the ability to reason and make inferences from observations using semantic technologies, giving access to the wider set of applications that makes use of the Semantic Web.
- It enables the straightforward use of querying mechanisms, such as SPARQL, to discover new information.
- It provides the possibility of integrating new observations with the great amount of information enabled through RDF and OWL in the Semantic Web. This point is closely related to the Linked Data concept introduced by Berners-Lee, which refers to “data published on the Web in such a way that it is machine-readable, its meaning is explicitly defined, it is linked to other external data sets, and can in turn be linked to from external data sets [34].”

**Figure 3.**
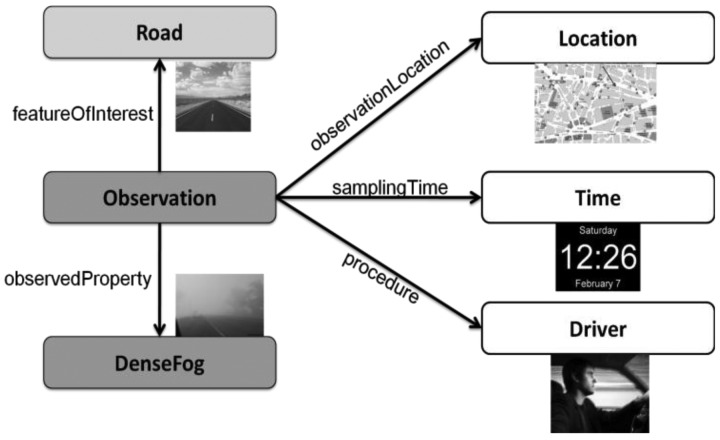
O&M-OWL model (adapted from [35]) applied to driver-generated observations.

In order to provide a semantic representation for the driver's observations, we have followed the approach proposed by Henson *et al.* [35] based on the encoding of the OGC Observations and Measurements language (O&M) in OWL (the Web Ontology Language [36]). In O&M-OWL an ontology covers a subset of concepts in O&M, and, similarly to what is proposed in [35] for general sensor observations, we think it can also offer interesting possibilities for managing human-generated observations. Figure 3 shows the translation of O&M into OWL, adapted to our driver-generated observations scenario; it is important to note that, as can be seen in the figure, the O&M property *procedure* (denoting the instrument, algorithm or process used to collect the observation) is the “driver”.

In O&M-OWL, relations between concepts are described using RDF triples, which correspond to a subject-predicate-object structure. As an example, the O&M-OWL representation of a driver-generated observation of the presence of dense fog on a road would be as listed in Table 1:

In this example it is important to point out that, as discussed in Section 3, the in-vehicle HMI system collects (by engaging in either driver-initiated or sensor-initiated dialogue) only the driver's description of the observed phenomenon (*i.e.*, the *om:featureOfInterest*). Consequently, the HMI architecture has to automatically provide all the remaining data to be included in the O&M-OWL representation describing the human-generated observation. This includes the particular situation of the car on the road (*om:observationLocation*) and the observation time (*om:samplingTime*). It is also important to note that, as shown in the example, the observation entity (*om:procedure*) can be linked to a particular driver or to an anonymous driver (or a nickname). This can be very useful when addressing the relevant issue of privacy management of human-generated observations (see the discussion and study in Section 6).

**Figure 4.**
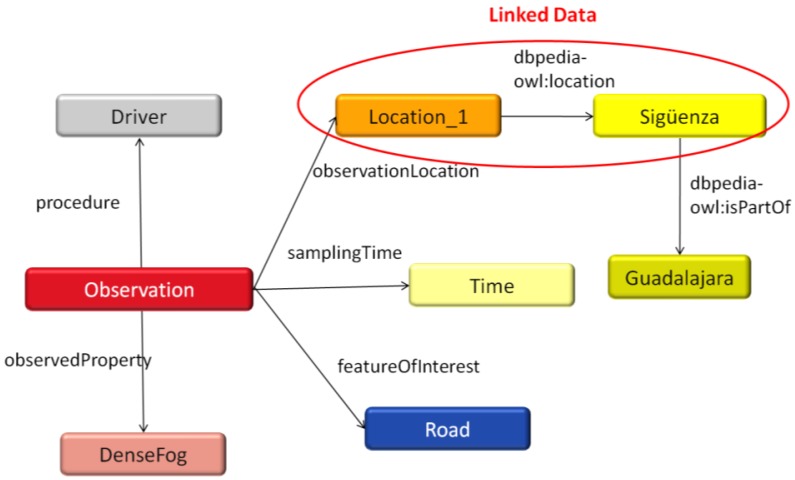
Driver-generated observations linked to DBpedia resources.

Moreover, as we stated at the beginning of this subsection, by using Linked Data principles data published on the Semantic Web can be reused in the sensor annotation procedure. This makes it possible to annotate sensor data by creating RDF links to other data from sources like DBpedia [37], which is more efficient and “shareable” than defining new ontologies with their corresponding concepts and relationships. To illustrate this, Figure 4 shows how, in our previous example, the O&M *location* value (*om:location_1*) can be linked to a specific location, “Sigüenza,” defined by DBpedia (*dbpedia:Sigüenza*) using the property *location* defined by the DBpedia ontology (*dbpedia-owl*). Furthermore, in DBpedia, the object “Sigüenza” is related to other objects. For example, “Sigüenza” is defined as part of another location named “Guadalajara” (see Figure 4). The flexibility and richness of structured information offered by Linked Data opens the possibility of performing advanced queries and inferences on these driver-generated observations, as we show in the following subsection.

### 4.2. Publishing on the Semantic Sensor Web

Together with the use of O&M-OWL to generate driver-generated observations encoded as a set of RDF triples, it is important to consider how these semantically annotated observations can be accessed for inference or query.

In our work, in contrast to the use of semantically enabled OGC services proposed in [35] (in particular the extension of SOS to SemSOS), we have explored a preliminary step towards making human-generated observations accessible using the existing information space of the Web. We stored RDF driver-observations in public repositories (*i.e.*, SPARQL Endpoints [38]). By doing so O&M-OWL observations encoded in RDF and linked to specific ontologies can be shared with other systems and applications through the Semantic Sensor Web (SSW). The information published in the SSW can then be used for a wide variety of purposes: it can be further mashed up with other information to acquire yet higher levels of knowledge, it can be pooled to analyze patterns of use of applications (for example for a Road Safety Authority), or it can be fed back to applications (e.g., other connected car HMI systems), thus closing an information loop, with a connected entity producing and consuming context-aware information.

To illustrate with an example, applications could have access to the human-generated observations stored as RDF Graphs, which can be retrieved via SPARQL queries. Through these queries it will be possible to filter the RDF triples in the repository that fulfill a set of desired conditions. The following example shows a SPARQL query searching for driver-generated observations from roads in a specific area, “Guadalajara,” and with “denseFog” as the observed property.

PREFIX environment:<http://www.sensor.gaps.upm.es/environment/>
PREFIX dbpedia:<http://dbpedia.org/resource/>
PREFIX om:<http://www.opengis.net/om/1.0>
PREFIX rdf:<http://www.w3.org/1999/02/22-rdf-syntax-ns#>
PREFIX dbpedia-owl:<http://dbpedia.org/ontology/>
SELECT DISTINCT ?obs WHERE {
  ?c rdf:type environment:Road .
  ?obs om:featureOfInterest ?c ;
    om:observedProperty environment:denseFog ;
    om:observationLocation ?loc .
  ?loc dbpedia-owl:isPartOf dbpedia:Guadalajara.
}

In this example the query works by matching the triples RDF in the “WHERE” clause against the triples in the RDF graph stored in the repository. Our RDF example in Section 4.1 matches this clause because the observation was linked to a specific location datum in the DBpedia domain. Thus, information available in the Semantic Web is reused. The observation was linked to the resource “Sigüenza,” which is related to the resource “Guadalajara” through the DBpedia ontology (by virtue of the *isPartOf* property”). Thus the query result may include the values corresponding to our particular observation (along with all the other observations published in the repository that may fulfill the query requirements). In this case the value (*om:obs_1*), that represents an observation from a specific road segment (*om:road_1*), would be assigned to the variable *?obs*.

Additional knowledge from semantically annotated driver-observations could be obtained by using rule-based reasoning to infer new ontological assertions from known instances and class descriptions. For example, the driver-generated observation of a road under dense foggy conditions in the previous subsection could be used by a Road Safety Authority monitoring application to warn other drivers entering the area. A driver planning a trip through this area using a navigator connected to the Semantic Sensor Web (in her car or mobile phone) could be alerted of the dense fog and be advised to take an alternative route.

## 5. Experimental Setup

We set up an experiment to perform an exploratory analysis of the different approaches and technologies we have considered for sharing driver-generated observations, collected using in-vehicle HMI systems, through the Semantic Sensor Web. Our testing scenario approximates the realistic connected car environment developed in the MARTA [12] (Mobility for Advanced Transport Networks) research project, in which several HMI systems were developed for different In-Vehicle Information Systems (IVIS) and Advanced Driver Assistance Systems (ADAS) applications.

The instantiation of the W3C MMI architecture described in Section 3, including mechanisms to annotate and publish driver-observations (Section 4), was carried out on an On-Board Unit (OBU) in charge of managing the Human-Machine Interaction. This OBU was integrated with the new technologies (GRPRS—General packet radio service, UMTS—Universal Mobile Telecommunications System, HDSPA—High Speed Downlink Packet Access, CALM—Continuous Air interface for Long and Medium distance, *etc*.) developed in MARTA to give support to V2V (vehicle to vehicle) and V2I (vehicle to infrastructure) communications.

The final implementation was integrated in a *CarPC*, which is a computer designed to be specifically installed and run in vehicles. The *CarPC* was set up with a Linux OS, a Java Virtual machine and release 3.4 of the OSGi platform [24].

**Figure 5.**
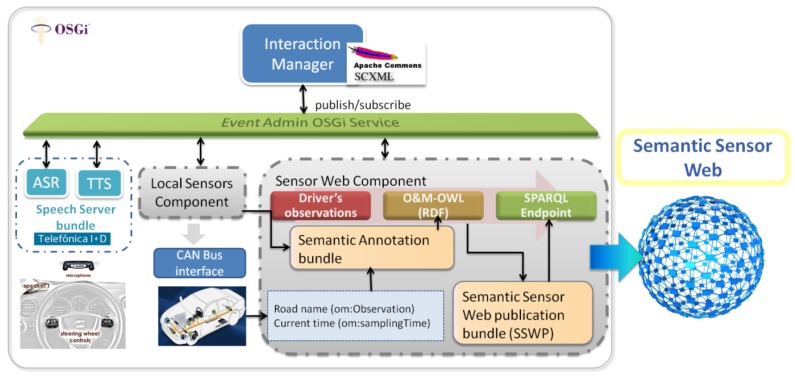
Experimental setup for publishing driver-generated observations in the Semantic Sensor Web.

Figure 5 presents the main components we developed on the vehicle side of our implementation. As described in Section 3, the Interaction Manager of our MMI architecture was implemented using SCXML, so a specific bundle was developed including the SCXML engine provided by Apache Commons SCXML [39]. Both the driver-initiated and the sensor-initiated dialogues were implemented using SCXML documents invoking proprietary Telefónica R&D speech technologies (ASR—Automatic Speech Recognition and TTS—Text to Speech) accessed through a Speech Server bundle. Dialogue management also included interaction with events from buttons on the steering wheel (which served to carry out functions such as allowing the driver to generate an order to start a driver-initiated dialogue).

The event-based communication layer (again, see Section 3)—an important element in the W3C MMI architecture—was supported by the EventAdmin OSGi Service bundle, which is a standard way of dealing with events using the publish/subscribe model. It is through this service that the SCXML-based Interaction Manager interacts with the Speech Server bundle (ASR/TTS) as well as with several bundles receiving sensor data (*i.e.*, Sensor Components). Data from Local Sensor Components (car-sensors) were received through specific wrapping components that accessed the CAN (Controller Area Network) bus, while a specific bundle, including Internet access through GPRS, was developed to access the Semantic Sensor Web (*i.e.*, to query RDF repositories).

**Figure 6.**
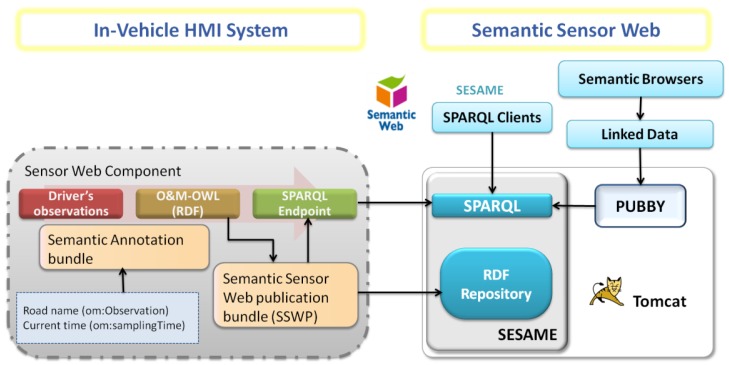
Connection between our Experimental Setup and the Semantic Sensor Web for publishing driver-generated observations.

Another important experimental development was the integration of several technologies to reach the final goal of making the driver-generated observations, collected through the SCXML dialogues, available on the Semantic Sensor Web. To this end we followed two major development steps:

- First, a specific bundle (the Semantic Annotation bundle in Figure 5) was implemented. This bundle receives events from the Interaction Manager and generates RDF annotations using the O&M-OWL model. As discussed in Section 4, to complete the data in all the generated RDF triples, this bundle was connected to other in-car information systems; in our case to the navigation system, to obtain the road name (*om:observation*), current time (*om:samplingTime*) and position (as the precise km on a particular road, *om:observationLocation*).
- Second, each time the Semantic Annotation bundle generates a RDF annotated driver-observation, a Semantic Sensor Web publication bundle (SSWP, see Figure 5) is used to publish it in a RDF repository. For this purpose we have made use of features provided by Sesame [40]: an open source Java Framework for the storage and querying of RDF data. More specifically, we have used the Sesame workbench to create an offline repository. Consequently, as shown in Figure 6, each time the SSWP bundle generates an RDF annotation, Sesame is used to add the corresponding new RDF triples into an RDF repository (more specifically into a SPARQL EndPoint [38]).

However, it is important to notice that driver-observations stored as RDF triples in repositories can be only accessed by sending SPARQL queries to a SPARQL endpoint. In RDF the resources are identified by means of URIs. These URIs used in the SPARQL repositories are not dereferenceable, meaning that they cannot be accessed from a Semantic Web Browser and therefore by a growing variety of Linked Data applications and clients. For example, in our particular car-related scenario the resources in the namespace *environment* (used in the example in Section 4) can be found following the URL *http://www.sensor.gaps.upm.es/environment/*. However, the SPARQL endpoint is accessible through the local address *http://www.sensor.gaps.upm.es/openrdf-sesame/repositories/environment*. Therefore, the RDF in this repository only will be accessible locally by the SPARQL clients, making it necessary to perform a mapping that allows access through semantic browsers and linked data clients.

To tackle this difficulty, Pubby [41], a Linked Data Front End for SPARQL Endpoints was integrated with our initial Sesame repository, as depicted in Figure 6. Pubby also provides a server (only requiring a servlet container such as Apache Tomcat) that is in charge of mapping the URIs retrieved by SPARQL endpoints to dereferenceable URIs. Pubby handles requests from semantic browsers by connecting to the SPARQL endpoint, requesting from it information regarding the original URI, and returning the results to the client through an access point. So, with the Pubby server configured to run at *http://www.sensor.gaps.upm.es/environment/*, when the semantic browser or linked data client decides to access a particular URI, such as *http://www.sensor.gaps.upm.es/environment/Road*, it accesses the Pubby server, which then collects the information regarding the resource in question from the SPARQL endpoint (*http://www.sensor.gaps.upm.es/openrdf-sesame/repositories/environment*). The resource information is then returned to the client in machine-readable format.

This, in sum, is how we are able to make the new driver-generated observations collected through in-vehicle HMI systems shareable over the Semantic Sensor Web.

## 6. Performance Analysis and Concept Validation

**Figure 7.**
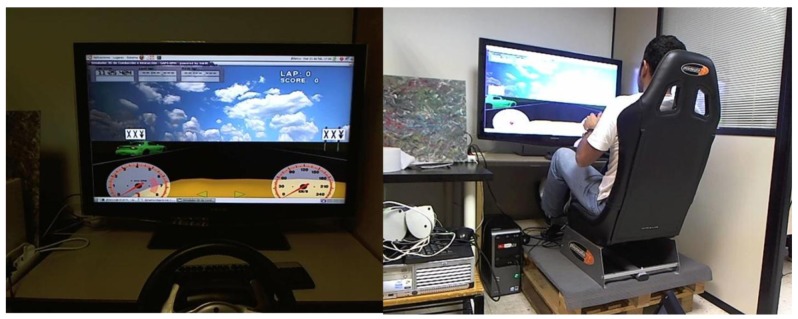
Driving simulator for performance analysis and usability evaluation following the Lane Change Test protocol.

In addition to the experimental setup implemented on an On-Board Unit, the same software components were integrated in a driving simulator environment (see Figure 7), so we could have a flexible and safe testing environment for performance analyses and usability studies.

The driving simulator was designed with the open-source driving simulator *VDrift* [42] (details of our implementation are presented in [43]). The driving simulator and the interaction framework were integrated through a standard connection in order to make contextual information available to the interaction framework. The HMI system consists of an application developed using OSGi and SCXML technologies, with which the driver can report a limited number of 16 observations (driver-initiated dialogue) or confirm a specific situation in a dialogue that is automatically initiated when a vehicle sensor (or set of sensors) detects a reportable situation such as a broken down car stopped on the side of the road (see Section 3). Performance tests and a conceptual validation of the scenario with potential users were carried out using this driving simulation framework.

### 6.1. Performance Analysis

A set of performance tests were carried out to guarantee proper response times within which to inform the HMI system of context changes. Response times were measured for varying numbers of concurrent contextual information sources (that could correspond to both sensors and Sensor Web sources), and for a varying degree of complexity of these sources.

Since our implementation is SCXML-based, our performance analysis focused on measuring response times for the SCXML machines involved in processing the events coming from different numbers of concurrent context sources, demanding SCXML processing with different complexities (*i.e.*, involving a different number of states). The test consisted in measuring the time elapsed from the arrival of a set of events to the SCXML structure, until their complete processing by this structure (*i.e.*, with the generation of a stable output). c (*x*) of events, simulating the arrival of *x* concurrent sensor observations, triggered the activation of *x* state machines, which in turn triggered a chain of transitions of a given number of states (*y*) representing the processing needs of these state machines.

**Figure 8.**
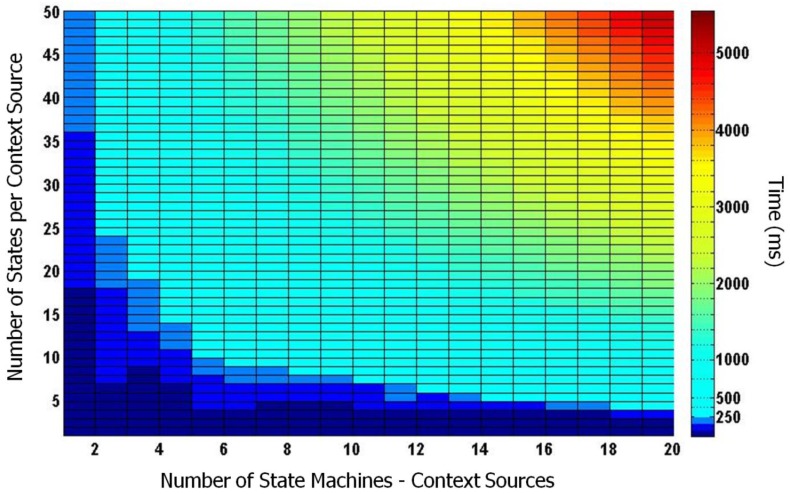
SCXML response times for different numbers of context sources of varying complexity (*i.e.*, number of states).

Simulation results are presented in Figure 8. The colormap in the figure corresponds to the SCXML processing response times in ms. on a grid with: 20 columns, for a variable number of concurrent events or context sources (*x* = 1 to 20); and 49 rows corresponding to a variable number of states (*y* = 2 to 50) corresponding to several possible processing demands for each one of the concurrent context sources. An SCXML state machine with 2 states can process a single sensor detecting a simple event, such as a low level of fuel (a state machine that only changes its state if the fuel level drops below a given threshold), while a machine with 50 states might be what would be needed to model the driver's steering behavior.

To analyze the performance results in Figure 8, we assumed that response times below 250 ms. were appropriate for the reactive behavior of an Interaction Manager in safety-critical situations, for example (as proposed in [44]) to suspend the interaction when the driver is carrying out a difficult maneuver in traffic. Within these low response times we found that our system was able to manage a variety of configurations, corresponding to the darker blue areas of Figure 8, ranging from around 20 context sources requiring low-complexity processing (less than 4 states), to a small number of sources (less than 4) demanding high processing (from 25 to 50 states).

Other areas of interest in Figure 8 (light blue and yellow) are covered by response times lower than 4 s. In these areas the information processed by the context sources can be used by the HMI Interaction Manager at the lower pace of turn exchanges with the user. Thus, for example, the HMI system can inform the driver that the observation she is reporting has already been confirmed by other drivers and already been communicated to local authorities. For these response times our performance analysis revealed that an average number of 18 context sources could be used demanding average processing load of 35 states each.

Above these areas our implementation handles a number of concurrent sources greater than 20 with complexities of over 35 states in processing times greater than 4 s. (red zone in the upper right corner of Figure 8). This performance area may be acceptable for deriving useful high-level contextual information that is relevant during the course of a journey, but not safety-critical nor 2-way interaction intensive (otherwise interaction with the driver would be badly interrupted, causing frustration and distraction).

### 6.2. Test Scenario Design and Validation

In the driving simulator we implemented the Lane Change Test (LCT) [45] in order to carry out quantitative and qualitative measures about driving performance degradation while the driver interacts with an interaction framework, and vice versa. In the upcoming tests, each driver will be involved in a driving task in which she had to keep a speed of 60 km/h along a 3-lane road. In addition, the test drivers will be instructed to keep in a specific lane indicated by signs that appear at regular intervals on both sides of the road (Figure 7). At the same time the drivers will be asked to execute a secondary task that is not restricted by the standard. In our case, we developed a task related to the publication of driver-generated observations through a HMI system following our approach described in this paper. Two kinds of such observations were considered: observations provided freely by the driver and observations recorded by the system (*i.e.*, generated passively by the driver). As mentioned previously (in Section 3), interactions will either be system-initiated or driver-initiated.

To provide some form of validation of the test scenario we produced a questionnaire designed to elucidate what kinds of information users might be willing to receive and to share with smart applications, in which contexts, and whether they would have concerns about the idea. We had 33 respondents, 22 male and 11 female; most with at least some driving experience, of which 24 declared they were either good or expert drivers. The questionnaire included items with a 5-point Likert response format, with anchors in the extremes (“strongly disagree,” assigned a value of −2, and “strongly agree,” assigned a value of 2); multiple choice questions; and the option to write comments for some of the questions. We now present some results from this preliminary validation based on the expectations of potential users.

**Figure 9.**
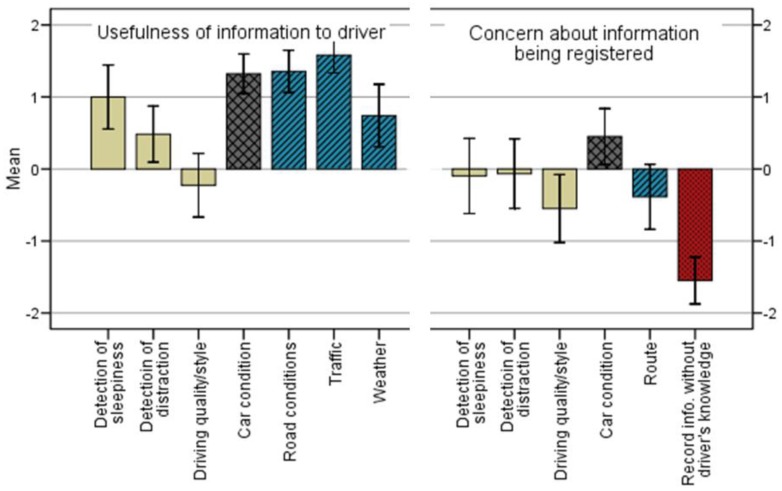
Means* of responses to items^†^ related to expected usefulness of different sources of information to the driver (left half of the figure) and to concerns about having information registered (and possibly shared) by the system (right half)^‡^. Notes: * 95% confidence intervals are shown; ^†^ Items are grouped by color/texture, denoting, from left to right, driver- (tan/plain), vehicle- (grey/crossed) and environment-related (blue/dashed) items; ^‡^ The higher the concern, the more negative the corresponding value. An extra item of concerns about having information registered without the driver's knowledge is shown in red.

To begin, we asked potential users whether they would regard useful a system that would give them information related to the three basic ontological dimensions of driving: the driver, the vehicle and the environment. Respondents were the most positive about the usefulness of being *consumers* of information from the driving environment (road conditions, weather and traffic), and the most skeptical about information concerning the driver. However, responses were widely varied across different kinds of information. The left half of Figure 9 shows the mean value of the responses for each of these items. Detection of sleepiness and distraction were, on average, better received than the monitoring of driving quality, the latter not being generally regarded as providing useful information. Furthermore, respondents expressed a variety of concerns (right half of Figure 9) regarding the collection of all such information and the prospect of it being shared with other parties (*i.e.*, with the focus on drivers as *producers* of information) except that which concerns the mechanical condition of the car (its positive value indicates lack of concern, on average). The more troubling sources of information were driving style and route planning (the latter shown in blue, as an environmental item, though it has a clear component of personal information about the driver). The respondents' comments revealed details of the concerns. The most common concern by far was privacy (11 respondents stated it), followed by fear that the information might be inappropriately used (7 respondents), reluctance on account of the expected increase in workload, stress and distraction (5 respondents), and feeling controlled (3 respondents). The greatest source of concern, in any case, was that the system might record information about the driver without his or her knowledge (red bar at the far right in Figure 9).

For none of the sources of information considered was there a correlation found between the expected usefulness of receiving it and the corresponding concerns associated with its collection by the system (*i.e.*, between the items on the left hand side of Figure 9—usefulness and the corresponding ones on the right hand side of Figure 9—concerns). This may suggest that there is a degree of independence between how useful people find services and the concerns they may have regarding how the information is collected and used, as indeed the nature of the information matters greatly also; or it could be revealing a relative independence of the potential users' willingness to be informed from their reluctance to have information about their driving behavior collected, even when the functioning of the services require collecting the information. Though more discriminative testing is needed to sort out these intricacies, respondents' comments reveal varying attitudes (which may account for the lack of correlation), from those who express interest in the scenario services and few concerns; to those who showed less interest while expressing reluctance due to privacy and other concerns; to those with little interest simply because they don't see how the information could be useful (regardless of privacy and other concerns).

When asked whether they would prefer the interaction initiative to fall entirely on the system, or on the driver, or whether they would prefer a mixed initiative scheme, the latter was clearly preferred (67% respondents expressed agreement with this preference). Interestingly, however, there were differences regarding opinions of the three initiative schemes depending on the driving situation. Specifically, we distinguished between driving in an urban area and driving on other roads (e.g., a motorway or country road). For urban areas we found that mixed initiative was thought to be the more comfortable interaction set-up by only slightly more respondents (39%) than driver (30%) or system initiative (24%); for other roads mixed initiative was clearly favored in terms of comfort (64%). System initiative was thought the safer mode of interaction in urban areas (49%), however, while on other roads the figures for system initiative were closer to those for mixed initiative (33% and 39% respectively). When asked which interaction set-up was expected to be the most distracting, mixed initiative was chosen the least (and, thus, overall we can infer that it was considered the least distracting mode) both for urban areas (12%) and other roads (18%).

The preference for mixed initiative can be taken as a first indication that users might be willing to contribute information voluntarily, feeling in control of the information provided. On the other hand, as mentioned above, potential users reject the thought of unknown information being recorded by the system. Personal information is very sensitive (and almost anything pertaining to the driver seems to be regarded as such), and confidence in the knowledge of what the information will be used for seems crucial for acceptance. The context of the driving activity also has to be taken into account. With these observations we are now in a better position to formulate a scenario of use combining the lane-change task with an appropriately designed secondary task (interaction through a dialogue system).

## 7. Conclusions and Further Research

The central theme presented in this paper has been that interconnected objects, embedding Human-Machine Interaction (HMI) technologies, can play an important role to obtain relevant human-generated real-world information that can be shared with other users, connected objects or applications.

As a particular connected object scenario, we have discussed how the design of in-vehicle HMI systems can make driver-generated observations shareable on the Semantic Sensor Web. Our approach is based on collecting observations from drivers using an HMI system built following the W3C MMI architecture, incorporating semantic annotation using O&M-OWL, and making the RDF-generated data available to SPARQL Endpoints and Linked Data Front-Ends. An experimental setup, integrating different HMI and Semantic Web technologies, implemented on an OSGi platform for a connected car On-Board Unit, has also been presented.

This experimental framework, upon which we are developing a test scenario for a “connected car”, has served to illustrate the possibilities of integrating HMI systems into emerging Sensor Web initiatives. It has also highlighted important challenges that need to be addressed, some related to HMI system development while others to the future evolution of the Sensor Web.

We have begun the validation of the test scenario that we are developing to look experimentally at driver-initiated and sensor-initiated dialogues. The approach seems sound, since, on the one hand, potential drivers believe the proposed driving-assistance systems in the scenario can be useful, and on the other, they show willingness to actively engage in conversation with the on-board HMI system, contributing information voluntarily. A great amount of care has to be taken, however, to provide users with a sense of control of the information that is being shared, especially personal information, including that describing driver behavior. It will be interesting to observe the effects of information sharing during the upcoming driving-interaction test-runs (using the Lane-Change Test mentioned in Section 6.2). We will also look at whether, and to what extent, inaccuracies in sensor information and limitations on the number of allowed reported observations generate frustration in drivers, and whether this leads to unsafe interaction patterns. Yet another focus of attention will be the impact of interaction problems, such as misunderstandings and non-understandings of user utterances, on driving quality, user acceptance and indeed the efficiency and limitations of using interaction as a source of sensed information.

The continual emergence of new terms such as Sensor Web, Real-World Internet, Semantic Sensor Web, Semantic Sensor Internet or Human Sensor Web, suggests that more fundamental research effort is required to advance in how to effectively articulate and provide access to human-generated observations, human sensors, sensor observations and the Internet. We believe that the use of Semantic Web principles and technologies can assist in this task, but as the amount of human-generated and sensor-generated data grows, scalability and efficiency for intensive distributed computing will become critical factors. Our future research will also address mechanisms for the proper management of privacy and quality of information, two key aspects for the successful sharing of human-generated observations.

## Figures and Tables

**Table 1.** O&M-OWL representation of a driver-generated observation of dense fog.

om:obs_1	rdf:type	om:Observation
om:obs_1	om:featureOfInterest	om:road_1
om:road_1	rdf:type	environment:Road
environment: Road	rdfs:subClassOf	om:Feature
om:obs_1	om:observedProperty	environment:denseFog
environment:denseFog	rdf:type	om:Property
om:obs_1	om:samplingTime	om:time_1
om:time_1	rdf:type	owl-time:Instant
om:time_1	owl-time:date-time	“20110610T08:55:00”
om:obs_1	om:procedure	om:human_1
om:human_1	rdf:type	environment:Driver
om:obs_1	om:observationLocation	om:location_1 .

(Explanatory note: *om* is used as a namespace for O&M and is placed, with a colon, before the concepts defined in the O&M schema; concepts from the environment ontology contain the namespace *environment*, and *dbpedia* represents a link from a location observation to a dbpedia URI).

## Abbreviations

ADAS: Advanced Driver Assistance Systems
ASR: Automatic Speech Recognition
CALM: Continuous Air interface for Long and Medium distance
CAN: Controller Area Network
CS: Catalogue Service
GPRS: General Packet Radio Service
HDSPA: High Speed Downlink Packet Access
HMI: Human Machine Interaction
IVIS: In-Vehicle Information Systems
LCT: Lane Change Test
MARTA: Mobility for Advanced Transport Networks
MMI: W3C's Multimodal Architecture and Interfaces
OBU: On-Board Unit
OGC: Open Geospatial Consortium
OSGI: Open Services Gateway Initiative
OWL: Web Ontology Language
O&M: Observation & Measurement
RDF: Resource Description Framework
SAN: Sensor and Actuator Network
SCXML: State Chart eXtensible Markup Language
SensorML: Sensor Model Language
SOS: Sensor Observation Service
SPARQL: SPARQL Protocol and RDF Query Language
SSW: Semantic Sensor Web
SWE: Sensor Web Enablement
TTS: Text to Speech
UMTS: Universal Mobile Telecommunications System
URI: Uniform Resource Identifier
URL: Uniform Resource Locator
V2I: Vehicle to Infrastructure
V2V: Vehicle to Vehicle
W3C: World Wide Web Consortium
